# From Fluoride Removal to Interfacial Reactivity Enhancement: Functionalized La^3+^‐Crosslinked B/CuO Energetic Microspheres

**DOI:** 10.1002/advs.77314

**Published:** 2026-08-20

**Authors:** Hongbiao Huo, Minghao Bao, Zhongbo Han, Wei Shi, Jianyong Xu, Pengfei Cui, Wenchao Zhang

**Affiliations:** ^1^ School of Chemistry and Chemical Engineering Nanjing University of Science and Technology Nanjing China; ^2^ State Key Laboratory of Transient Chemical Effects and Control of China Shaanxi Applied Physics and Chemistry Research Institute Xi'an China

**Keywords:** adsorption‐functionalization coupling, B/CuO energetic microspheres, combustion performance enhancement, fluoride‐containing wastewater, interfacial fluoride immobilization

## Abstract

The treatment of fluoride‐containing wastewater is essential for environmental protection. Although some studies have investigated fluoride recovery following adsorption, current research has primarily focused on fluoride removal and adsorbent regeneration, and the subsequent utilization of recovered fluoride as a functional component remains largely unexplored. Herein, an integrated strategy is proposed by coupling fluoride removal with the performance regulation of energetic materials. Based on the sodium alginate‐La^3+^ crosslinking strategy, the as‐fabricated B/CuO composite microspheres effectively immobilize fluoride ions at the interface, which subsequently act as functional modifiers to enhance combustion performance. A maximum capacity of 0.965 mg/g and 96.5% removal were achieved at 10 g/L (10 mg/ L F^−^). Structural characterizations reveal selective association with La‐based sites, while the material framework remains intact. Compared with the pristine B/CuO composite microspheres, the fluoride‐loaded microspheres exhibit more concentrated heat release, a 32.4% increase in average crushing strength, a 42% shorter burning duration, and markedly reduced combustion product size. By demonstrating that fluoride ions recovered from wastewater can directly enhance combustion performance, this work provides a new strategy for transforming recovered fluoride into a functional component for energetic materials.

## Graphical abstract

A fluoride adsorption‐functionalization strategy directly couples fluoride wastewater remediation with combustion performance enhancement of B/CuO energetic microspheres. Fluoride captured from wastewater is retained within the microspheres without adsorbent regeneration or disposal, simultaneously achieving pollutant removal and enhanced energetic performance.

## 1 Introduction

Water pollution caused by emerging contaminants has become a major global environmental challenge. Various classes of pollutants, including pharmaceutical residues [1], synthetic dyes [2], antibiotics [3, 4], and inorganic ions [5], have attracted extensive attention because of their persistence, ecological risks, and potential threats to human health. Consequently, a variety of treatment technologies, such as adsorption, membrane separation, advanced oxidation, and electrochemical processes, have been extensively investigated for wastewater remediation. Fluoride is widely present in industrial wastewater discharged from sectors such as chemical processing, metallurgy, electronics, and battery manufacturing. Its excessive release has become a critical issue threatening ecological systems and human health [6, 7, 8]. The long‐term accumulation of fluoride ions (F^−^) in the human body can lead to dental fluorosis, skeletal fluorosis, and even neurological damage [9, 10]. Current technologies for treating fluoride‐containing wastewater primarily include precipitation [11, 12], ion exchange [10, 13], membrane separation [14, 15], electrochemical methods [16, 17], and adsorption [18, 19]. However, existing studies have predominantly focused on the efficient removal of fluoride ions and the regeneration performance of adsorbents, while largely overlooking the subsequent utilization of the adsorbed species. This limitation not only results in low resource utilization efficiency but may also lead to the generation of secondary waste.

Boron (B) and copper oxide (CuO) are selected as a representative fuel‐oxidizer pair due to their high energy density, favorable thermochemical properties, and widespread use in delay charges and thermite systems [20, 21]. However, the surface of boron is inherently covered by a native B_2_O_3_ layer (∼2–5 nm thick), which impedes mass and heat transfer, thereby limiting the combustion efficiency and prolonging the reaction time of the energetic composite [22, 23]. Introducing fluorine has been shown to be an effective strategy for enhancing the combustion performance of boron [24, 25, 26, 27].

Accordingly, if fluoride ions present in fluoride‐containing wastewater can be judiciously reutilized and converted into combustion‐promoting components within boron‐based energetic materials, it would be possible to simultaneously achieve wastewater purification and enhanced combustion performance, thereby establishing a synergistic “waste‐to‐value” strategy. However, a survey of the existing literature reveals a clear disconnect between the two fields [28]: wastewater treatment research has predominantly focused on the efficient removal of fluoride ions and adsorbent regeneration, whereas the energetic materials community typically relies on externally introduced fluorinated additives. Effective integration between these domains remains largely unexplored. To date, no studies have directly employed fluoride ions captured from wastewater to regulate the combustion behavior of energetic materials. Consequently, the precise immobilization and functional conversion of wastewater‐derived fluoride into active, combustion‐promoting species remain unresolved technical challenges.

To address the aforementioned challenges, while also improving the interfacial contact between fuel and oxidizer and enhancing structural uniformity and processability, B/CuO energetic composite microspheres were fabricated via a sodium alginate‐La^3+^ ionic crosslinking strategy, enabling the homogeneous dispersion and stable encapsulation of fuel and oxidizer at the microscale. The as‐prepared microspheres were further employed as adsorbents for the removal of fluoride ions from aqueous solutions. More importantly, the adsorbed fluoride ions were subsequently utilized to regulate the interfacial structure and combustion behavior of the energetic microspheres. A systematic investigation was conducted to elucidate the adsorption performance, structural evolution, and combustion characteristics of the microspheres before and after fluoride adsorption. This work proposes an “adsorption‐functionalization” strategy that not only achieves efficient fluoride removal but also enhances the combustion performance of energetic materials, establishing a pathway to directly harness recovered fluoride ions and transform waste streams into value‐added functional resources, thereby offering a new paradigm for environmentally oriented material design.

## 2 Results and Discussion

### 2.1 Preparation and Structural Characterization of Energetic Composite Microspheres

The overall experimental workflow employed in this study is presented in Figure 1. B/CuO energetic composite microspheres were prepared via a sodium alginate‐La^3+^ ionic crosslinking strategy, followed by fluoride adsorption in simulated fluoride‐containing wastewater, determination of fluoride concentration before and after adsorption, and subsequent performance evaluation, including pressing, ignition, and combustion product characterization. The detailed microsphere formation process is schematically illustrated in Figure S2. During gelation, La^3+^ ions diffuse from the external solution into the alginate droplets and coordinate with carboxyl groups, thereby constructing a three‐dimensional crosslinked network. This network enables the effective encapsulation and homogeneous confinement of boron (B) and copper oxide (CuO) particles at the microscale. Scanning electron microscopy (SEM) observations reveal that the as‐prepared microspheres exhibit a well‐defined spherical morphology (as shown in Figure 2e1) with a narrow particle size distribution (as shown in Figure S3), indicating the high stability and reproducibility of the fabrication process.

**FIGURE 1:**
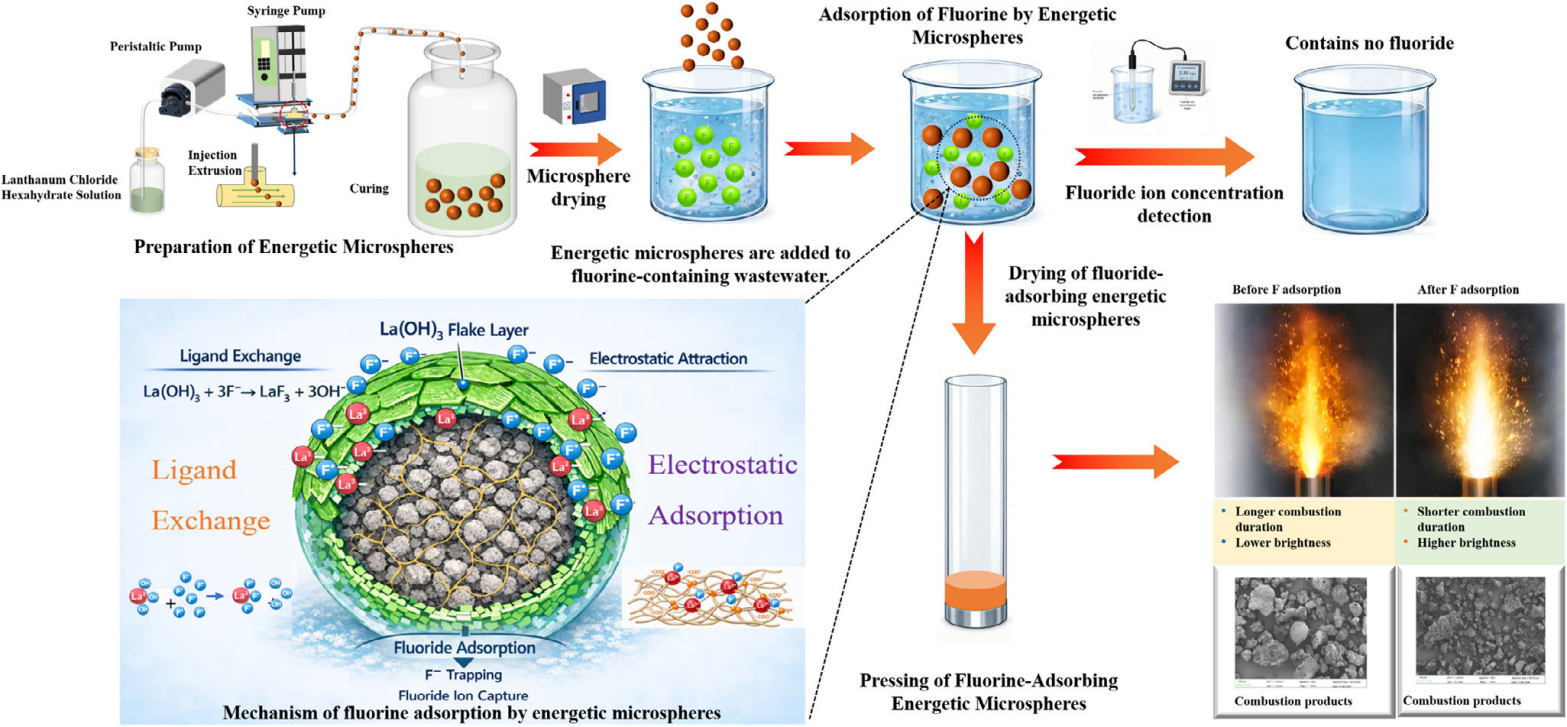
Schematic illustration of the preparation, fluoride adsorption, pressing, ignition, and combustion product characterization of energetic composite microspheres. The adsorption‐related conceptual illustration was initially generated using GPT‐5.5 (accessed April 2026) to provide a preliminary draft, followed by modifications, refinements, and annotations by the authors using Microsoft PowerPoint. All content was reviewed and finalized by the authors.

**FIGURE 2:**
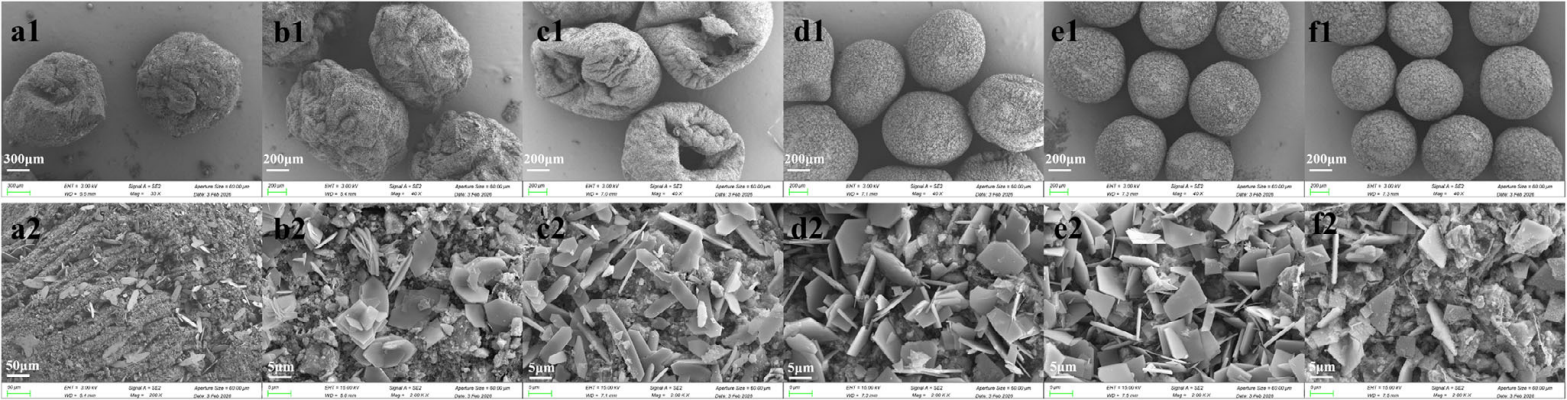
Effect of binder content on energetic composite microsphere morphology: (a1–e1) SEM images of energetic composite microspheres with binder contents of 1%–5%; (a2–e2) SEM images of the corresponding energetic composite microsphere surfaces; (f1) SEM image of energetic composite microspheres after fluoride adsorption; (f2) SEM image of the surface of energetic composite microspheres after fluoride adsorption.

Notably, the binder content (sodium alginate) plays a critical role in regulating the structural integrity and morphological evolution of the microspheres, as shown in Figure 2a1–e1. At relatively low alginate concentrations, insufficient network formation results in poor structural stability, leading to surface wrinkling or even the collapse of the microspheres. With increasing alginate content, a continuous and robust three‐dimensional crosslinked framework is established. This framework not only stabilizes the spherical morphology but also provides a confined microenvironment for ion diffusion and interfacial reactions. Meanwhile, an increase in binder content is accompanied by a gradual rise in the density of flake‐like structures on the microsphere surface, suggesting that the formation of surface crystals is closely associated with both the degree of crosslinking network development and the extent of interfacial ion enrichment.

Interestingly, the microspheres exhibit distinct structural heterogeneity, characterized by flake‐like features on the outer surface while maintaining a dense, uniform internal framework (as shown in Figure 2a2–e2 and Figure S4). This phenomenon indicates a typical interface‐dominated formation mechanism. Under diffusion‐controlled crosslinking conditions, La^3+^ ions preferentially accumulate in the outer regions of the microspheres. This distribution was further confirmed by EDS elemental mapping results shown in Figure S5. The formation of surface flake‐like structures is closely correlated with the presence of nanoscale CuO particles. Due to the hydroxylated surface and weak alkalinity of CuO [29], a localized alkaline microenvironment is induced at the microsphere interface, which promotes the hydrolysis of La^3+^ ions and subsequently facilitates the formation of layered La(OH)_3_ precipitates. This hypothesis is further supported by comparative experiments: no such flake‐like structures are observed in the pure boron system, whereas pronounced surface crystallization appears in CuO‐containing samples (as shown in Figure S6).

### 2.2 Fluoride Adsorption Behavior and Structural Stability

The fluoride adsorption performance of the energetic composite microspheres was first systematically evaluated, and the corresponding results are presented in Figure 3, with detailed analyses provided in the Supporting Information (Figures S7–S10 and Tables S1 and S2). The adsorption kinetics exhibit a rapid initial uptake followed by a gradual approach to equilibrium. This behavior is well described by the pseudo‐second‐order kinetic model (R^2^≈0.999), indicating that the adsorption process is predominantly chemisorption‐driven. Further isotherm analysis reveals that the Langmuir model (R^2^> 0.99) provides a better fit than the Freundlich model, suggesting that fluoride adsorption mainly occurs on homogeneous surface sites with monolayer coverage. Under an initial fluoride concentration of 10 mg/L and an adsorbent dosage of 10 g/L, the maximum adsorption capacity and removal efficiency reach 0.965 mg/g and 96.5%, respectively, demonstrating the excellent fluoride removal capability of the microspheres.

**FIGURE 3:**
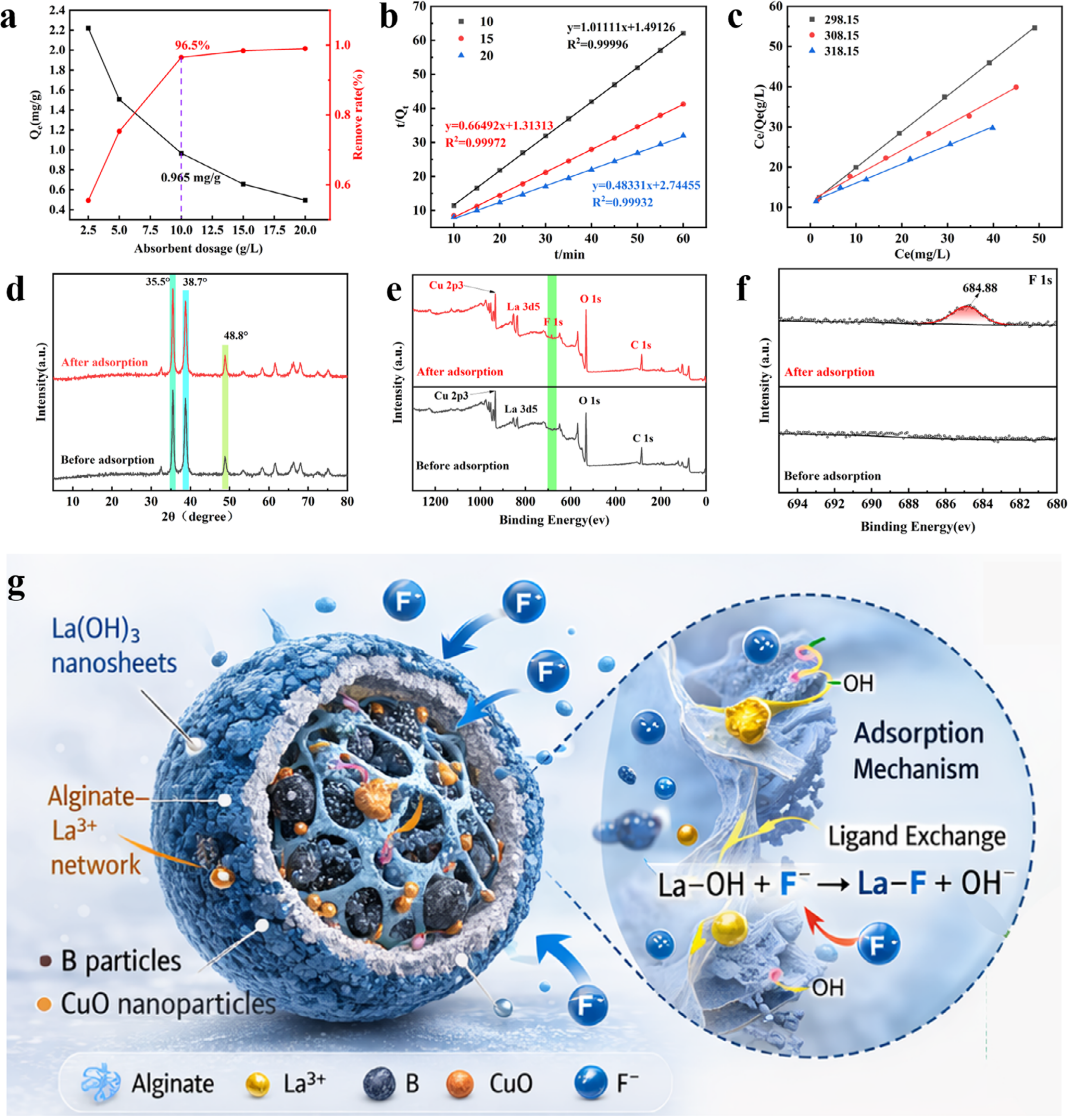
Adsorption behavior, kinetics, isotherm analysis, and structural characterization of energetic composite microspheres before and after fluoride adsorption: (a) Effect of energetic composite microsphere dosage on F^−^ adsorption; (b) Pseudo‐second‐order kinetic curves; (c) Langmuir fitting curves; (d) XRD patterns; (e) XPS survey spectra; (f) F 1s spectra. (g) Schematic illustration of the fluoride adsorption mechanism. The schematic in (g) was initially generated using GPT‐5.5 (accessed April 2026) to obtain a preliminary draft, followed by modifications, refinements, and annotations performed by the authors using Microsoft PowerPoint. The final schematic was reviewed and finalized by the authors.

Based on the adsorption performance, the morphological evolution of the microspheres before and after fluoride adsorption was further examined. As shown in Figure 2e2–f2, the pristine microspheres exhibit a relatively smooth, layered surface structure. After fluoride adsorption, the surface becomes significantly roughened, accompanied by the formation of numerous fine particles and localized etching features. This morphological transformation indicates that fluoride ions interact with surface active sites, inducing interfacial reconstruction and the formation of new interfacial species. Despite these surface changes, the overall spherical morphology of the microspheres remains intact, suggesting that the adsorption process is primarily confined to the interfacial region without compromising the macroscopic structural integrity.

To further elucidate the structural evolution at the molecular and crystalline levels, comprehensive FTIR, XRD, and XPS characterizations were conducted, as shown in Figure S11 and Figure 3. The FTIR spectra display consistent characteristic peaks at 3360, 1590, 1415, 1091, and 1048 cm^−1^ before and after adsorption, indicating that the alginate backbone and its coordination with La^3+^ remain essentially unchanged. Similarly, no new diffraction peaks or noticeable peak shifts are observed in the XRD patterns, confirming that the overall crystalline structure of the system is preserved. These results collectively demonstrate that the adsorption process does not disrupt the bulk framework but is predominantly associated with interfacial regulation.

XPS analysis further provides insight into the binding mechanism of fluoride ions. After adsorption, the La 3d peaks exhibit a shift toward lower binding energy (as shown in Figure S12), indicating a modification of the local electronic environment. Meanwhile, the appearance of the F 1s signal confirms the successful incorporation of fluoride species into the system (as shown in Figure 3e,f). Combined analysis suggests that fluoride ions are immobilized at La‐based coordination sites via a ligand‐exchange mechanism (La‐OH → La‐F), rather than by simple physical adsorption. In contrast, the Cu and C spectra show negligible changes, indicating that this interaction is primarily localized at the interface and does not perturb the bulk composition.

Overall, fluoride ions undergo a transition from rapid capture to stable binding within the system, achieving interface‐selective structural regulation. This process enables effective interfacial functionalization without compromising the structural integrity of the microspheres, thereby providing a critical structural foundation for subsequent performance enhancement.

### 2.3 Thermal Behavior, Flowability, Bulk Density, and Mechanical Properties of Microspheres

The thermal decomposition behavior of the microspheres, both before and after fluoride adsorption, was investigated by differential scanning calorimetry (DSC) under an inert atmosphere. The results obtained at a heating rate of 10°C/min are presented in Figure 4, while those at other heating rates are provided in Figure S13. As shown in Figure 4a, the decomposition peak temperature of the pristine energetic composite microspheres is 659.20°C, which decreases to 656.23°C after fluoride adsorption. Notably, the decomposition peaks of the fluoride‐modified microspheres shift to lower temperatures, indicating enhanced sensitivity to external thermal stimuli and an overall increase in reactivity. In addition to the peak shift, the exothermic peaks of the fluoride‐containing microspheres are more concentrated and sharper than those of the pristine system, suggesting a more synchronous and intensified reaction. This behavior can be attributed to modifications in the fuel‐oxidizer interfacial chemistry induced by surface fluorine species. On the one hand, fluorine may participate in interfacial activation by weakening the stability of the native B_2_O_3_ oxide layer, thereby facilitating the exposure of active boron sites [24, 30]. On the other hand, the formation of fluoride‐containing intermediates may alter the melting behavior and mass transport characteristics during the reaction, thus accelerating the overall reaction kinetics [31, 32]. Collectively, the introduction of surface fluorine enhances the interfacial reactivity of the B/CuO composite microspheres, as evidenced by the lower decomposition temperature and more pronounced heat release.

**FIGURE 4:**
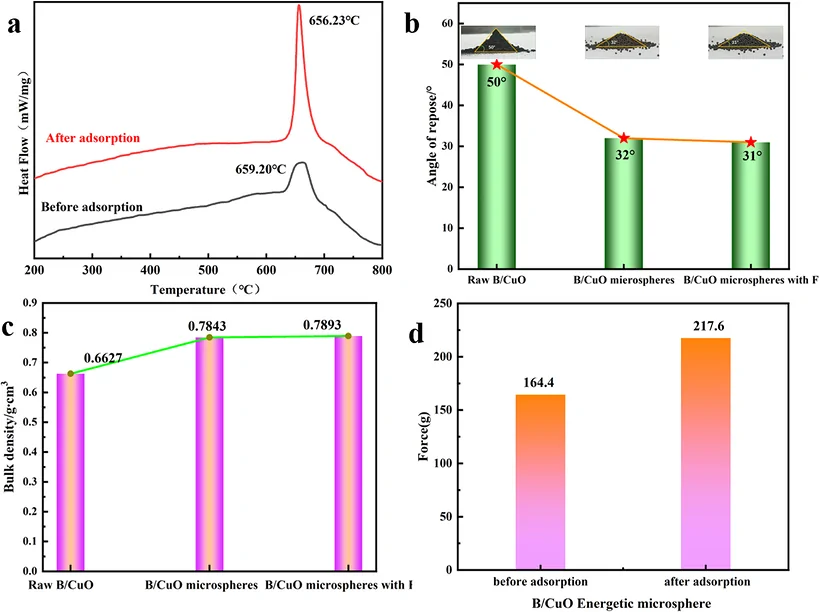
Thermal behavior, flowability, bulk density, and mechanical properties of energetic composite microspheres: (a) DSC curves; (b) Flowability; (c) Bulk density; (d) Mechanical properties.

The formation of microspheres also significantly improves the macroscopic handling properties of the energetic materials, as shown in Figure 4b,c. The angle of repose decreases markedly from 50° for the raw B/CuO powder to 32° for the microspheres, indicating a substantial enhancement in flowability (Figure S14). After fluoride adsorption, the angle of repose remains essentially unchanged (31°), demonstrating that the structural integrity of the microspheres is well preserved. Similarly, the bulk density increases from 0.6627 g/cm^3^ for the raw powder to 0.7843 g/cm^3^ after microsphere formation, which can be attributed to the reduced interparticle voids and improved packing efficiency. A slight further increase to 0.7893 g/cm^3^ after fluoride adsorption confirms that the adsorption process does not compromise the structural stability of the material (Table S3). In addition, the mechanical strength of the microspheres was evaluated using a texture analyzer. Three independent measurements were performed for each sample, and the corresponding force‐time curves are presented in Figure S15. The average crushing force obtained from these measurements is summarized in Figure 4d. Before fluoride adsorption, the B/CuO microspheres exhibited an average crushing force of 164.4 g. After fluoride adsorption, the average crushing force increased to 217.6 g, representing an approximately 32.4% increase, indicating improved mechanical load‐bearing capability. This improvement can be attributed to the gradual conversion of some La‐OH groups into La‐F bonds during fluoride adsorption. Owing to the higher bond energy and greater stability of La‐F bonds, the inorganic framework within the microspheres becomes more robust, thereby enhancing their mechanical strength.

These results indicate that the microsphere architecture effectively transforms the powder system into a more processable and densely packable form, which is highly advantageous for practical applications such as compaction and charge loading.

### 2.4 Combustion Behavior and Performance Enhancement

To more realistically simulate combustion behavior under practical operating conditions, a fixed amount of energetic composite microspheres was compacted into quartz tubes to construct a confined charge structure [33, 34], followed by ignition tests to systematically evaluate their combustion characteristics, as shown in Figure 5. Each sample was tested in triplicate to ensure reliability and reproducibility, and the corresponding results are shown in Figures S16 and S17. A direct comparison between the pristine and fluoride‐modified samples reveals pronounced differences in reaction dynamics and flame evolution pathways. For the microspheres without fluoride adsorption, ignition leads to the formation of a slender flame channel with relatively low luminosity. The flame propagates slowly along the axial direction but exhibits evident instability during propagation, including flame oscillation, localized ejection, and intermittent intensification. The flame front appears discontinuous and fluctuating, suggesting that the reaction interface is constrained by mass and heat transfer limitations. As a result, energy release occurs intermittently, leading to a prolonged combustion duration and a non‐uniform burn rate distribution. In contrast, the fluoride‐modified charge exhibits a distinctly different combustion behavior. Upon ignition, the flame rapidly intensifies, forming a bright, continuous, and axially stable flame column. The combustion process becomes more concentrated and uniform, with minimal observable attenuation or fluctuation. Notably, the total combustion duration is reduced by approximately 42%, and the reaction front propagates more steadily, indicating a substantial enhancement in both reaction activity and burning rate.

**FIGURE 5:**
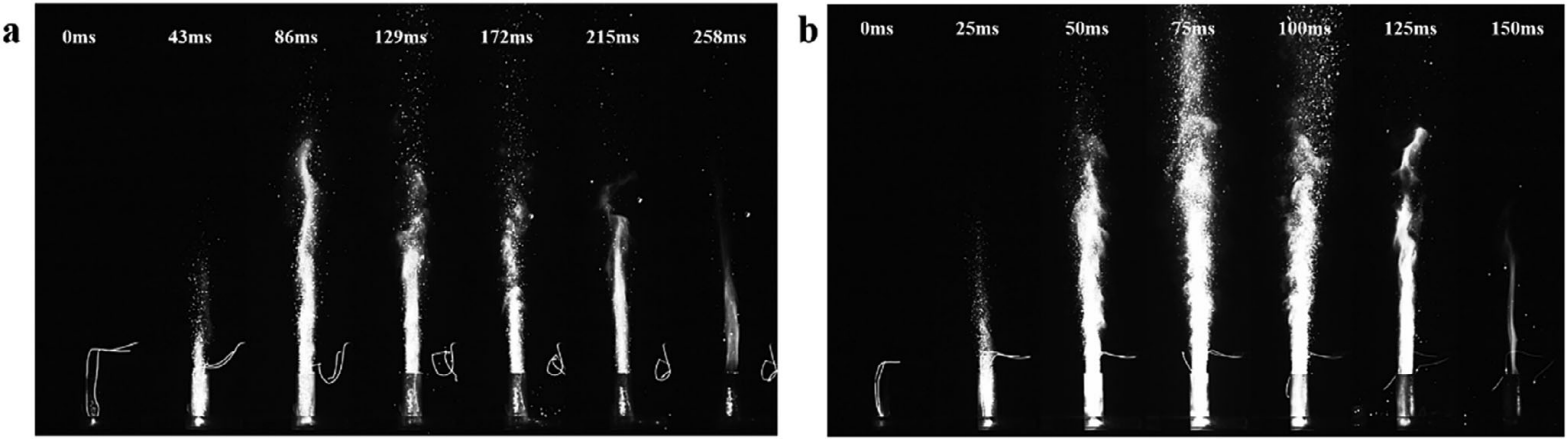
High‐speed imaging of the combustion behavior of pressed energetic composite microspheres: (a) Before adsorption; (b) After adsorption.

These differences are further corroborated by the morphology of the combustion residues (as shown in Figure S18). The pristine sample leaves behind a large number of coarse agglomerated particles, with evident particle coalescence and partially melted, resolidified dense structures. Residual large fragments are still observable, indicating incomplete reaction and the presence of localized “reaction dead zones” due to insufficient interfacial contact. In contrast, the fluoride‐modified sample exhibits more refined, more uniformly distributed residues, with markedly reduced agglomeration. The overall structure becomes loose and porous, and large dense remnants are nearly absent, indicating a more complete and homogeneous reaction process.

By correlating the combustion behavior with residue evolution, it can be inferred that fluorine plays a critical role in interfacial regulation within the system. During the initial stage of combustion, fluorine preferentially participates in interfacial reactions, weakening the stability of the B_2_O_3_ oxide layer on boron and facilitating the exposure of active boron sites. Meanwhile, the formation of low‐viscosity or volatile fluorine‐containing intermediates during combustion effectively reduces the coverage and blockage of the reaction interface by molten products, thereby significantly improving mass and heat transfer conditions. Through this synergistic mechanism, the reaction mode transitions from an initially “intermittent propagation” regime to a “continuous propagation” regime, ultimately manifesting as brighter flames, enhanced combustion stability, and accelerated reaction kinetics at the macroscopic scale.

## 3 Conclusion

In this study, B/CuO energetic composite microspheres were successfully fabricated using a sodium alginate‐La^3^^+^ ionic crosslinking system combined with a self‐built setup, achieving uniform integration and stable immobilization of the fuel and oxidizer at the microscale. Adsorption studies indicate that the fluoride adsorption behavior of the composite microspheres is better described by the Langmuir isotherm model, with correlation coefficients exceeding 0.99. This suggests that the adsorption predominantly follows a monolayer mechanism on a homogeneous surface, and the theoretical maximum adsorption capacity shows good agreement with the experimental results. Flowability measurements demonstrate that the microspherization process significantly improves the macroscopic physical properties of the particulate system. Specifically, the angle of repose decreases from 50° for the raw powder to approximately 32°, while the bulk density increases from 0.6627 g/cm^3^ to about 0.78 g/cm^3^. These results indicate that particle sphericity and optimized particle size distribution effectively enhance both flowability and packing efficiency. Mechanical property measurements further demonstrate that the average crushing strength of the microspheres increases by 32.4% after fluoride adsorption, indicating that fluoride incorporation contributes to improved mechanical stability without compromising the structural integrity of the microspheres. Combustion experiments show that, after fluoride adsorption, the charge exhibits a more concentrated and stable combustion process, with significantly enhanced flame luminosity and a reduction in combustion duration of approximately 42%. Meanwhile, the combustion residues display pronounced particle refinement and reduced agglomeration, indicating a more complete reaction within the system.

These findings demonstrate that this adsorption‐functionalization strategy enables the direct conversion of recovered fluoride into a functional component of energetic materials, thereby achieving fluoride wastewater remediation and enhanced combustion performance. Compared with conventional adsorbents that require post‐use regeneration or disposal, this strategy eliminates secondary treatment and directly uses the captured fluoride to improve the combustion performance of energetic materials, offering a new approach to integrating environmental remediation with the development of multifunctional energetic materials.

## Author Contributions

**Minghao Bao**: conceptualization, formal analysis, validation. **Wenchao Zhang**: conceptualization, writing – review and editing, resources, funding acquisition, supervision. **Zhongbo Han**: formal analysis, methodology. **Hongbiao Huo**: conceptualization, methodology, formal analysis, writing – review and editing, writing – original draft, data curation, validation. **Wei Shi**: formal analysis, investigation. **Pengfei Cui**: conceptualization, writing – review and editing, formal analysis, supervision. **Jianyong Xu**: formal analysis, investigation.

## Conflicts of Interest

The authors declare no conflicts of interest.

## Supporting information




**Supporting File**: advs77314‐sup‐0001‐SuppMat.docx.

## Data Availability

The data that support the findings of this study are available from the corresponding author upon reasonable request.
