# Supplementary material for: From Fluoride Removal to Interfacial Reactivity Enhancement: Functionalized La3+‐Crosslinked B/CuO Energetic Microspheres
Source: Adv Sci (Weinh). 2026 Aug 20:e77314. Online ahead of print. doi: 10.1002/advs.77314 (PMC13491525; doi:10.1002/advs.77314)
Supplement: Supplementary file 1 [file ADVS-9999-e77314-s001.docx]

**Supporting Information**

**From Fluoride Removal to Interfacial Reactivity Enhancement: Functionalized La^3+^-Crosslinked B/CuO Energetic Microspheres**

*Hongbiao Huo ^a^, Minghao Bao ^a^, Zhongbo Han ^a^, Wei Shi ^a^, Jianyong Xu ^a^,* *Pengfei Cui ^a,*^,* *Wenchao Zhang ^a,b*^*

*^a^ School of Chemistry and Chemical Engineering, Nanjing University of Science and Technology, Nanjing 210094, China*

*^b^ State Key Laboratory of Transient Chemical Effects and Control of China, Shaanxi, Applied Physics and Chemistry Research Institute, Xi’an, 710061, China*

**Corresponding Author E-mail address:*

*[pfcui@njust.edu.cn](mailto:pfcui@njust.edu.cn)(Pengfei Cui)，zhangwenchao@njust.edu.cn (Wenchao Zhang)*

**4. Experiment**

4.1 Experimental Materials

Boron powder (1-3μm), Jiangsu Zhiren Jingxing New Materials Research Institute Co., Ltd.; Copper oxide (40nm), Shanghai Maclean Biochemical Technology Co., Ltd.; Sodium alginate, Shanghai Aladdin Biochemical Technology Co., Ltd.; Lanthanum chloride hexahydrate, Shandong Keyuan Biochemical Co., Ltd.; Sodium fluoride, Shanghai Aladdin Biochemical Technology Co., Ltd.; Deionized water, prepared in the laboratory.

4.2 Screening of B/CuO ratio

To investigate the effect of the mass ratio of boron powder (B) to copper oxide (CuO) on the heat of combustion of the system, three different ratios were set up (B to CuO mass ratios of 5:95, 10:90, and 15:85), and the heat of combustion (unit: J/g) of the system under each ratio was tested. The results are shown in **Figure S1**. When the mass ratio of B to CuO was 5:95, the heat of combustion of the system was 2485.63 J/g; when the ratio was increased to 10:90, the heat of combustion increased significantly to 2780.75 J/g; while when the ratio was further increased to 15:85, the heat of combustion decreased slightly to 2734.72 J/g. It can be seen that the heat of combustion of the system reaches its peak under the condition of a B to CuO mass ratio of 10:90, indicating that this ratio is more conducive to improving the energy release level of the system.

Figure S1. Effect of boron/copper oxide ratio on the exothermic properties

4.3 Preparation of Energetic Composite Microspheres

An optimized mixture of boron powder and copper oxide was added to an aqueous solution of sodium alginate and uniformly dispersed under stirring and sonication. The resulting solution was then transferred to a 10 ml syringe, and microspheres were prepared using a self-built device. Given the high adsorption capacity of lanthanum for fluoride ions [1,2], an aqueous solution of lanthanum chloride hexahydrate was selected as the pipeline flow system. The specific preparation process is shown in **Figure S2**.

Figure S2. Schematic diagram of the preparation process for energetic compo site microspheres.

4.4 Adsorption Test

4.4.1 Preparation of Fluoride Ion Solution Concentration

100 mg/L NaF Stock Solution: Dry NaF at 105℃ for 2 h, cool to room temperature, weigh 0.2210 g, dissolve in 1000 mL of deionized water, and store in a polyethylene bottle for later use. Fluoride solutions of different concentrations will be obtained by diluting the stock solution.

4.4.2 Adsorption process

0.4 g of energetic composite microspheres were weighed and placed in a 50 ml centrifuge tube containing 40 ml of 10 mg/L fluoride solution. The pH was not adjusted, and the reaction was carried out at room temperature with shaking at 200 rpm for 6 h. After filtration through a filter membrane, the fluoride ion concentration was measured.

In the adsorption kinetics experiment, solutions with initial fluoride concentrations of 10, 15, and 20 mg/L were used to investigate the effect of different fluoride concentrations on the adsorption rate. In the adsorption isotherm experiment, solutions with initial fluoride concentrations of 10, 20, 30, 40, 50, and 60 mg/L were used at different temperatures (25℃, 35℃, and 45℃) to investigate the adsorption performance of the energetic composite microspheres for fluoride. The fluoride adsorption capacity of this adsorbent material was predicted using a model.

4.4.3 Calculations for the Adsorption Experiment

The adsorption capacity of energetic composite microspheres per unit mass for fluoride ions, $q_{e}$(mg/g), is calculated using equation (1).

$q_{e}=\frac{\left(C_{0}-C_{e}\right)\cdot v}{m}$ （1）

Where $C_{0}$ and $C_{e}$ are the initial concentration of the fluorine solution and the concentration at adsorption equilibrium, respectively, in mg/L; $V$ is the volume of the fluorine solution, in L; and $m$ is the amount of adsorbent added, in g.

4.4.4 Adsorption Kinetics Calculation

Adsorption kinetics studies evaluate the adsorption rate of the adsorption process, qualitatively analyze the adsorption type, and ultimately explore the adsorption mechanism of pollutants by the adsorbent. Data obtained from kinetic experiments are used to fit kinetic equation models, allowing the adsorption process to be described by relevant parameters obtained from the model fitting. Adsorption kinetics research provides crucial data support for the application of energetic composite microspheres in fluoride-containing wastewater. This experiment employs quasi-first and second-order kinetic equation models to study the adsorption mechanism of fluoride by energetic microspheres.

The formulas for its first-order and second-order dynamic equations are shown in equations (2) and (3).

First-order dynamic equation formula:

$\ln\left(Q_{e}-Q_{t}\right)=\ln Q_{e}-K_{1}t$ （2）

Second-order dynamic equation formula:

$\frac{t}{Q_{t}}=\frac{1}{K_{2}Q_{e}^{2}}+\left(\frac{1}{Q_{e}}\right)t$ （3）

In the formula: $Q_{e}$ is the adsorption capacity of the adsorbent for F^-^ at equilibrium (mg/g), $Q_{t}$is the adsorption capacity of the adsorbent for F^-^ at reaction time t (mg/g), t is the reaction time (min), $K_{1}$is the first-order rate constant (1/min), and $K_{2}$is the second-order rate constant (g/(mg/min)).

4.4.5 Calculation of Adsorption Isotherms

The isothermal adsorption curve is the relationship between the adsorption capacity of the adsorbent and the equilibrium concentration of the adsorbate in the solution. The equilibrium adsorption capacity reflects the adsorption performance of the adsorbent, and isothermal parameters help explain the adsorption mechanism. The Langmuir and the Freundlich isothermal adsorption models were used to analyze the isothermal adsorption data. Origin 2023 software was used to perform nonlinear fitting on the adsorption isotherm and thermodynamic experimental data. The adsorption mechanism was analyzed by fitting the Freundlich and Langmuir adsorption isotherm models to the adsorption process at different temperatures. The Freundlich and Langmuir isotherm equations are as follows:

(1) Langmuir adsorption isotherm model

The Langmuir model is based on monolayer adsorption and the adsorbent having homogeneous adsorption sites, and the calculation method is shown in Equation (4).

Model mathematical expression:

$\frac{q_{e}}{q_{\max}}=\frac{K_{L}C_{e}}{1+K_{L}C_{e}}$ （4）

Linearized form:

$\frac{C_{e}}{q_{e}}=\frac{1}{K_{L}q_{\max}}+\frac{C_{e}}{q_{\max}}$ （5）

(2) Freundlich adsorption isotherm model

The Freundlich adsorption isotherm model is a nonlinear empirical model based on heterogeneous surfaces, used to analyze multilayer adsorption processes on heterogeneous surfaces. The calculation formula is shown in equation (6):

$q_{e}=K_{F}C_{e}^{1/n}$ （6）

Linearized form:

$\log q_{e}=\log K_{F}+\frac{1}{n}\log C_{e}$ （7）

Where: $C_{e}$—equilibrium concentration (mg/L);$q_{e}$—equilibrium adsorption capacity (mg/g);

$q_{max}$—theoretical maximum adsorption capacity (mg/g);$K_{L}$—Langmuir equilibrium adsorption constant;$K_{F}$—Freundlich equilibrium adsorption constant; n—empirical constant

4.5 Characterization

The surface morphology of the samples was observed using a Carl Zeiss Sigma 360 field emission scanning electron microscope (Carl Zeiss, Germany) with an accelerating voltage of 5 kV and a working distance of 8 mm. Before testing, the samples were platinum-coated for 60 s using an ion sputtering instrument (JEOL JFC-1600) to improve conductivity, and the images were acquired in secondary electron (SE) mode.

Phase analysis was performed using a Bruker D8 Advance X-ray diffractometer (Germany), with Cu Kα radiation (λ=1.5406 Å), a scan range of 5-80°, a step speed of 0.05°/s, a tube voltage of 40 kV, and a tube current of 40 mA. Data were processed using Jade 6.5 software and compared with PDF standard cards.

The differential scanning calorimeter (DSC) was tested using an HCT-4 model manufactured by Beijing Hengjiu Experimental Equipment Co., Ltd. (China). The sample (5 mg) was placed in an alumina crucible and heated from 25 °C to 1000 ℃ at constant rates of 10, 15, and 20 K/min under an argon atmosphere (flow rate 50 mL/min). An empty crucible was used as a reference. Temperature and heat flux calibration were performed before testing using high-purity indium (melting point 156.6 °C) and zinc (melting point 419.5 °C). The heat flux curves were analyzed using the instrument’s software, with endothermic peaks pointing upwards.

Fourier transform infrared (FTIR) spectroscopy was performed using a BRUKER (Germany) INVENIO spectrometer, scanning in the range of 4000-400 cm^-1^ with a resolution of 4 cm^-1^ and 32 scans. Sample preparation was performed using the ATR method, and data were processed using OPUS software.

Fluoride ion concentration was determined using a PXS-F fluoride ion meter manufactured by Hangzhou Qiwei Instrument Co., Ltd. (China). Instrument calibration employed a two-point method: the electrode slope was calibrated sequentially using 1.0 mg/L and 100.0 mg/L sodium fluoride (NaF) standard solutions. Before calibration, the electrodes were rinsed with deionized water until the blank potential value (≤±2 mV) was reached. During sample testing, the temperature was maintained at 25±0.5℃, and the magnetic stirring speed was 200 rpm. Data was recorded after the reading stabilized (change <0.5 mV within 30 s). Each sample was measured three times.

The combustion process was recorded using a high-speed camera (model: MEMRECAM ACS-1) manufactured by NAC Image Technology, Japan. A 0.4g powder sample was uniformly filled into a quartz tube, pressed into shape, and then ignited by applying electricity through a Ni-Cr wire. During combustion, the high-speed camera captured the dynamic behavior of flame propagation at a sampling frequency of 5000fps (frames per second) and simultaneously recorded key parameters such as flame morphology and spread speed.

Elemental chemical state analysis was performed using a Thermo Scientific ESCALAB 250Xi X-ray photoelectron spectrometer (USA), monochromaticized Al Kα radiation (hν = 1486.6 eV), with a pass energy set to 30 eV and a vacuum level better than 1 × 10^-9^ mbar. The C 1s peak (284.8 eV) was used as the binding energy correction reference during the test. Data were processed using Avantage software and combined with the NIST database for peak shape fitting and elemental valence state analysis.

The texture of the microspheres was analyzed using a British SMS TA.XT Plus texture analyzer. A 2 mm-diameter cylindrical probe was selected, and the test speed was set to 0.05 mm/s. Three parallel tests were performed on the same batch of samples to obtain the hardness curves of the composite microspheres.

**2 Results and Discussion**

**2.1 Preparation and Structural Characterization of Energetic Composite Microspheres**

Figure S3. Particle size distribution of energetic composite microsphere

Figure S4. Cross-sectional SEM image of energetic composite microsphere

Figure S5. EDS spectra and elemental mapping of La^3+^-crosslinked B/CuO energetic composite microspheres.

Figure S6. Optical microscope images of single-component microspheres of boron powder (a) and copper oxide (b).

**2.2 Fluoride Adsorption Behavior and Structural Stability**

**2.1.1 Effect of Adsorbent Dosage on Fluoride Adsorption**

The dosage of adsorbent plays a critical role in determining both the number of available adsorption sites and the overall adsorption capacity, thereby exerting a direct influence on fluoride removal efficiency. In this study, 0.1, 0.2, 0.4, 0.6, and 0.8 g of adsorbent were introduced into 40 mL of fluoride solution with an initial concentration of 10 mg/L to evaluate the effects of adsorbent dosage on the adsorption capacity and defluoridation efficiency of the energetic composite microspheres. The corresponding results are presented in **Figure S7**. As shown in **Figure S7**, the removal efficiency increases rapidly at low adsorbent dosages and gradually levels off with increasing dosage. This behavior can be attributed to the initially high fluoride concentration, where the limited number of active sites on the adsorbent exhibits a strong positive correlation with fluoride uptake. As the number of available active sites increases, the removal efficiency correspondingly improves. Considering that both adsorption capacity and removal efficiency are key parameters for evaluating adsorption performance, an adsorbent dosage of 10 g/L was selected as the optimal condition, under which the adsorption capacity reaches 0.965 mg/g, and the fluoride removal efficiency attains 96.5%. With further increases in adsorbent dosage, the gradual rise in removal efficiency is primarily attributed to the significantly reduced residual fluoride concentration in solution, which diminishes the mass transfer driving force between fluoride ions and the active adsorption sites.

Figure S7. Effect of energetic composite microsphere dosage on F^-^ adsorption

**2.1.2 Effect of Initial Concentration on Fluoride Adsorption**

Adsorption time is a critical parameter governing the overall adsorption process. This is not only because adsorbents with shorter equilibrium times are more favorable for practical industrial applications, but also because the variation in adsorption efficiency and capacity with time provides important insights into the adsorption mechanism [3].To evaluate the time-dependent adsorption performance of the energetic composite microspheres, the residual fluoride concentration was monitored at different adsorption times (0-60 minutes) for fluoride solutions with initial concentrations of 10 mg/L, 15 mg/L, and 20 mg/L. The corresponding results are presented in **Figure S8**. As shown in **Figure S8**, the adsorption rate is rapid within the first 15 minutes, which can be attributed to the relatively high concentration of fluoride ions (F^-^) in solution and the abundance of available active sites on the surface of the microspheres, facilitating efficient interaction between the two. Consequently, fluoride ions are quickly removed from the solution during this stage. Thereafter, the adsorption rate gradually decreases and eventually reaches equilibrium. In addition, the adsorption capacity at any given time increases with increasing initial fluoride concentration.

Figure S8. Effect of adsorption time on F^-^ adsorption at different fluoride ion concentrations.

**2.1.3 Adsorption Kinetics Analysis**

To provide a theoretical interpretation of the adsorption process, adsorption kinetics models were employed to simulate the time-dependent behavior and elucidate the underlying rate-controlling mechanisms [4]. The experimental data were analyzed using both pseudo-first-order and pseudo-second-order kinetic models. For the pseudo-first-order model, ln($Q_{e}-Q_{t}$) was plotted against time (t), as shown in **Figure S9**(a). For the pseudo-second-order model, t/$Q_{t}$ was plotted against t, as presented in **Figure S9**(b), and the corresponding kinetic parameters are summarized in **Table S1**. As indicated in **Table S1** and **Figure 8**, the pseudo-second-order model exhibits higher correlation coefficients (R^2^ = 0.99996, 0.99972, and 0.99932) compared to the pseudo-first-order model (R^2^ = 0.953, 0.961, and 0.988), demonstrating that the pseudo-second-order model is more suitable for describing the adsorption of F^-^ onto the energetic composite microspheres. Moreover, the equilibrium adsorption capacities calculated from the pseudo-second-order model ($Q_{e(cal)}$= 0.989 mg/g, 1.504 mg/g, and 2.069 mg/g) are in closer agreement with the experimentally measured values ($Q_{e(exp)}$ = 0.966 mg/g, 1.454 mg/g, and 1.875 mg/g), further confirming the validity of this model. **Table S1** also shows that an increase in the initial fluoride concentration leads to a higher adsorption capacity. However, the pseudo-second-order rate constant (K_2_) decreases from 0.686 to 0.085 with increasing initial fluoride concentration, indicating a gradual reduction in the adsorption rate at higher concentrations. Overall, the adsorption of F^-^ onto the energetic composite microspheres is dominated by a chemisorption process, in which chemical interactions serve as the rate-limiting step.

Figure S9. Pseudo-first-order (a) and pseudo-second-order (b) kinetic curves for F^-^ adsorption onto energetic composite microspheres at different initial concentrations.

Table S1. Pseudo-first-order and pseudo-second-order kinetic parameters for F^-^ adsorption by energetic composite microspheres

| Initial Fluoride Concentration | $Q_{e(exp)}$ (mg/g) | (Pseudo-first-order) | | | (Pseudo-second-order) | | |
| --- | --- | --- | --- | --- | --- | --- | --- |
|  |  | K_1_ | $Q_{e(cal)}$ | R^2^ | K_2_ | $Q_{e(cal)}$ | R^2^ |
| 10 | 0.966 | 0.082 | 0.270 | 0.953 | 0.686 | 0.989 | 0.99996 |
| 15 | 1.454 | 0.075 | 0.168 | 0.961 | 0.337 | 1.504 | 0.99972 |
| 20 | 1.875 | 0.086 | 1.561 | 0.988 | 0.085 | 2.069 | 0.99932 |

**2.1.4 Adsorption Isotherms**

In this study, the adsorption behavior of fluoride ions on the energetic composite microspheres was analyzed using both the Langmuir and Freundlich isotherm models. The Langmuir model describes monolayer adsorption on a homogeneous adsorbent surface, whereas the Freundlich model assumes adsorption on a heterogeneous surface. The experimental data obtained at different temperatures were fitted using Equation (4) for the Langmuir isotherm and Equation (6) for the Freundlich isotherm. The corresponding results are presented in **Figure S10** and **Table S2**. At all investigated temperatures, the correlation coefficients of the Langmuir model (R^2^ = 0.99982, 0.99613, and 0.99605) are consistently higher than those of the Freundlich model (R^2^ = 0.93819, 0.98155, and 0.95809), and all exceed 0.95. This indicates that the adsorption process of fluoride ions onto the energetic composite microspheres is better described by the Langmuir isotherm model. Furthermore, the theoretical maximum adsorption capacities derived from the Langmuir model show closer agreement with the experimental values under different temperature conditions. These results collectively demonstrate that the adsorption of fluoride ions on the energetic composite microspheres follows a homogeneous monolayer adsorption mechanism.

Figure S10. Freundlich (a) and Langmuir (b) fitting curves of energetic composite microspheres at 298.15K, 308.15K, and 318.15K.

Table S2. Isotherm fitting parameters for fluoride ion adsorption by energetic composite microspheres.

| T/K | Langmuir | | | Freundlich | | |
| --- | --- | --- | --- | --- | --- | --- |
|  | $q_{max}$ | $K_{L}$ | $R^{2}$ | $K_{F}$ | n | $R^{2}$ |
| 298.15 | 1.116 | 0.082 | 0.99982 | 0.895 | 10.464 | 0.93819 |
| 308.15 | 1.595 | 0.054 | 0.99613 | 0.892 | 5.360 | 0.98155 |
| 318.15 | 2.165 | 0.040 | 0.99605 | 0.934 | 4.059 | 0.95809 |

Figure S11. Fourier-transform infrared (FTIR) spectra of energetic microspheres before and after fluoride adsorption.

Figure S12. Fine elemental spectra of the energetic composite microspheres before and after adsorption.

**2.3 Thermal Behavior, Flowability, Bulk Density, and Mechanical Properties of Microspheres**

As shown in **Figure S13**, the energetic microspheres without fluoride adsorption exhibit decomposition peak temperatures of 659.20, 661.65, and 662.15 °C at heating rates of 10, 15, and 20 °C/min, respectively. The gradual shift of the peak temperature toward higher values with increasing heating rate is consistent with the typical kinetic behavior of non-isothermal decomposition processes. In contrast, the fluoride-adsorbed energetic microspheres display lower decomposition peak temperatures of 656.23, 656.44, and 656.59 °C under the same heating conditions. The overall shift of the peak temperature to lower values indicates an enhanced sensitivity of the decomposition process to external thermal stimulation.

Figure S13. Comparison of thermal properties of energetic composite microspheres before and after adsorption: (a) Before adsorption; (b) After adsorption

Figure S14. Comparison of the flowability of (a) unprocessed B/CuO powder, (b) B/CuO energetic microspheres, and (c) B/CuO energetic microspheres after fluorine adsorption.

Table S3. Bulk density of samples

| Samples | Mass/g | Bulk density/$g\cdot{cm}^{3}$ | Average bulk density/  $g\cdot{cm}^{3}$ |
| --- | --- | --- | --- |
| Raw B/CuO | 0.0663 | 0.663 | 0.6627 |
|  | 0.0667 | 0.667 |  |
|  | 0.0658 | 0.658 |  |
| B/CuO microspheres | 0.0788 | 0.788 | 0.7843 |
|  | 0.0780 | 0.780 |  |
|  | 0.0785 | 0.785 |  |
| B/CuO microspheres with fluorine adsorption | 0.0796 | 0.796 | 0.7893 |
|  | 0.0782 | 0.782 |  |
|  | 0.0790 | 0.790 |  |

Figure S15. Force-time curves of the energetic composite microspheres before and after fluoride adsorption: (a) Before adsorption; (b) After adsorption.

**2.4 Combustion Behavior and Performance Enhancement**

Figure S16. The combustion time of pressed energetic composite microspheres: (a) Before adsorption; (b) After adsorption.

Figure S17. High-speed imaging of the combustion behavior of pressed energetic composite microspheres: (a) Before adsorption; (b) After adsorption.

Figure S18. SEM images of the combustion products (a: before adsorption; b: after adsorption).

**References**

[1]	S. Jian, J. Liu, J. Wu, W. Yang, L. Ran, J. Hu, G. Duan, H. Yang, J. Ma, S. Jiang, Glutaraldehyde-crosslinked chitosan microspheres encapsulating La(OH)_3_ by electrospraying for efficient remediation of fluoride ions, Chem. Eng. J. 512 (2025) 162470. https://doi.org/10.1016/j.cej.2025.162470.

[2]	X. Zhou, B. Li, Q. Zhao, Effective removal and adsorption mechanism of fluoride from water by biochar-based Ce(III)-La(III)-crosslinked sodium alginate hybrid hydrogel, Int. J. Biol. Macromol. 272 (2024) 132925. https://doi.org/10.1016/j.ijbiomac.2024.132925.

[3]	E. Kumar, A. Bhatnagar, M. Ji, W. Jung, S.-H. Lee, S.-J. Kim, G. Lee, H. Song, J.-Y. Choi, J.-S. Yang, B.-H. Jeon, Defluoridation from aqueous solutions by granular ferric hydroxide (GFH), Water Res. 43 (2009) 490–498. https://doi.org/10.1016/j.watres.2008.10.031.

[4]	S. Ghorai, K.K. Pant, Equilibrium, kinetics and breakthrough studies for adsorption of fluoride on activated alumina, Sep. Purif. Technol. 42 (2005) 265–271. https://doi.org/10.1016/j.seppur.2004.09.001.
